# Cytotoxic effects of 15d-PGJ2 against osteosarcoma through ROS-mediated AKT and cell cycle inhibition

**DOI:** 10.18632/oncotarget.1704

**Published:** 2014-01-21

**Authors:** Chueh-Chuan Yen, Chung-Der Hsiao, Wei-Ming Chen, Yao-Shan Wen, Yung-Chan Lin, Ting-Wei Chang, Fang-Yi Yao, Shih-Chieh Hung, Jir-You Wang, Jen-Hwey Chiu, Hsei-Wei Wang, Chi-Hung Lin, Tain-Hsiung Chen, Paul Chih-Hsueh Chen, Chien-Lin Liu, Cheng-Hwai Tzeng, Jonathan A. Fletcher

**Affiliations:** ^1^ Division of Hematology and Oncology, Department of Medicine, Taipei Veterans General Hospital, Taipei, Taiwan; ^2^ National Yang-Ming University School of Medicine, Taipei, Taiwan; ^3^ Therapeutical and Research Center of Musculoskeletal Tumor, Taipei Veterans General Hospital, Taipei, Taiwan; ^4^ Epidermal Stem Cell Lab, Department of Bioscience Technology, Chung Yuan Christian University, Chung-Li, Taiwan; ^5^ Department of Orthopedics and Traumatology, Taipei Veterans General Hospital, Taipei, Taiwan; ^6^ Stem Cell Laboratory, Department of Medical Research and Education, Taipei Veterans General Hospital, and Institute of Pharmacology, Faculty of Medicine, National Yang-Ming University, Taipei, Taiwan; ^7^ Institute of Clinical Medicine, National Yang-Ming University School of Medicine, Taipei, Taiwan; ^8^ Institute of Traditional Medicine, National Yang-Ming University, Taipei, Taiwan; ^9^ Division of General Surgery, Department of Surgery, Taipei Veterans General Hospital, and Department of Surgery, Cheng-Hsin General Hospital, Taipei, Taiwan; ^10^ Institute of Microbiology and Immunology, and Cancer Research Center & Genome Research Center, National Yang-Ming University, Taipei, Taiwan; ^11^ Department of Education and Research, Taipei City Hospital, Taipei, Taiwan; ^12^ Department of Pathology and Laboratory Medicine, Taipei Veterans General Hospital, Taipei, Taiwan; ^13^ Department of Pathology, Brigham and Women's Hospital, Boston, MA, U.S.A

**Keywords:** 15d-PGJ2, AKT, PLK1, osteosarcoma

## Abstract

Polo-like kinase 1 (PLK1), a critical cell cycle regulator, has been identified as a potential target in osteosarcoma (OS). 15-deoxy-Δ12, 14-prostaglandin J2 (15d-PGJ2), a prostaglandin derivative, has shown its anti-tumor activity by inducing apoptosis through reactive oxygen species (ROS)-mediated inactivation of v-akt, a murine thymoma viral oncogene homolog, (AKT) in cancer cells. In the study analyzing its effects on arthritis, 15d-PGJ2 mediated shear-induced chondrocyte apoptosis via protein kinase A (PKA)-dependent regulation of PLK1. In this study, the cytotoxic effect and mechanism underlying 15d-PGJ2 effects against OS were explored using OS cell lines. 15d-PGJ2 induced significant G2/M arrest, and exerted time- and dose-dependent cytotoxic effects against all OS cell lines. Western blot analysis showed that both AKT and PKA-PLK1 were down-regulated in OS cell lines after treatment with 15d-PGJ2. In addition, transfection of constitutively active AKT or PLK1 partially rescued cells from 15d-PGJ2-induced apoptosis, suggesting crucial roles for both pathways in the anti-cancer effects of 15d-PGJ2. Moreover, ROS generation was found treatment with 15d-PGJ2, and its cytotoxic effect could be reversed with N-acetyl-l-cysteine. Furthermore, inhibition of JNK partially rescued 15d-PGJ2 cytotoxicity. Thus, ROS-mediated JNK activation may contribute to apoptosis through down-regulation of the p-Akt and PKA-PLK1 pathways. 15d-PGJ2 is a potential therapeutic agent for OS, exerting cytotoxicity mediated through both AKT and PKA-PLK1 inhibition, and these results form the basis for further analysis of its role in animal studies and clinical applications.

## INTRODUCTION

Primary bone tumors account for 0.2% of all malignancies. Osteosarcoma (OS) is the major tumor type [1]. Sequential neoadjuvant chemotherapy, surgery and adjuvant chemotherapy provide a of 5-year overall survival of 60−70% in patients with localized disease [2-4]. However, 40−50% of patients with initially localized disease develop recurrence during treatment [5,6], and less than 20% of newly diagnosed cases present with metastatic disease [3,7]. Most of these patients will eventually die of the disease due to refractoriness to therapy [2].

Previous studies have revealed several genetic mechanisms in OS tumorigenesis, including dysfunction of tumor suppressor mechanisms, such as cell cycle regulatory genes (e.g., *tumor protein 53* [TP^53^], *retinoblastoma 1* [*RB^1^*] and *mouse double minute 2 homolog* [*MDM^2^*]) [8-10] as well as novel tumor suppressor genes (e.g., *limbic system-associated membrane protein* [*LSAMP*]) [11-13]. In addition, telomere dysfunction [14,15], dysregulation of cell death and cytokine pathways [16,17] and upregulation of ezrin (EZR) have been reported in metastatic OS [18,19]. However, genetic complexity is a hallmark of high-grade OS, and currently, no ideal target has been identified.

Recently, two independent groups have identified polo-like kinase 1 (PLK1), a serine/threonine kinase that regulates many stages of mitosis and maintains genomic stability [20], as a potential target for OS treatment, using short hairpin RNA (shRNA) libraries in lentiviral vectors for screening of protein kinases [21,22]. In addition, previous studies have demonstrated the potential oncogenic role of PLK1 [23-26]. Therefore, inhibition of PLK1 could represent an effective treatment for OS.

15-deoxy-Δ12, 14-prostaglandin J2 (15d-PGJ2) has gained attention as a potential cancer treatment because of its unique anti-tumor activity. Earlier studies showed that it could significantly inhibit cell growth and induce apoptosis in cancer cells through activation of PPARγ [27-29]. However, recent evidence indicates that its cytotoxicity is largely PPARγ-independent, and is closely related to reactive oxygen species (ROS) generation [30-32], with subsequent inhibition of critical pathways, such as v-akt murine thymoma viral oncogene homolog (AKT) [33-37]. Interestingly, in arthritis, 15d-PGJ2 mediated shear-induced chondrocyte apoptosis via protein kinase A (PKA)-dependent regulation of PLK1 [38]. However, the role of 15d-PGJ2 in modulating PLK1 has never been explored in cancer. Since PLK1 has been identified as a potential target of OS, the role of 15d-PGJ2 in the treatment of OS deserves further investigation. In this study, we demonstrated that 15d-PGJ2 could exert a cytotoxic effect on OS cell lines via a ROS-mediated dual inhibition of AKT and the cell cycle.

## RESULTS

### 15d-PGJ2 inhibits growth and induces apoptosis of OS cell lines

**Figure 1:**
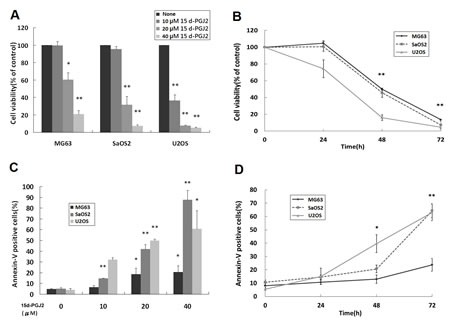
15d-PGJ2 inhibited the growth and induced apoptosis of OS cell lines (A, B) MTT assay. MG63, SaOS2 and U2OS cells were seeded 1 day before treatment with (A) various concentrations of 15d-PGJ2 (0, 10, 20, or 40 μmol/L) for 72 h or (B) 15d-PGJ2 (20, 10, 10 μmol/L) for the indicated time. Cell viability was assessed using the MTT assay and expressed as a percentage of viability under controlled culture conditions. (C, D) Apoptosis assay. MG63, SaOS2 and U2OS cells were seeded 1 day before treatment with (C) various concentrations of 15d-PGJ2 (0, 10, 20, or 40 μmol/L) for 72 h or (D) 15d-PGJ2 (20, 10, 10 μmol/L) for the indicated time. The percentage of apoptotic cells was determined using Annexin V-FITC/propidium iodide (PI) staining. All data represent the mean ± SD of three independent experiments. *P < 0.05; **P < 0.01.

To evaluate the effect of 15d-PGJ2 on the growth of OS cells, three OS cell lines (MG63, SaOS2, U2OS) were treated with various concentrations of 15d-PGJ2 for a range of time. Cell viability was assessed by 3-(4,5-dimethylthiazol-2-yl)-2,5-diphenyltetrazolium bromide (MTT; Sigma-Aldrich) assay. As shown in Figure 1A and 1B, 15d-PGJ2 significantly inhibited the growth of all three OS cell lines in a dose- and time-dependent manner.

We then investigated whether 15d-PGJ2 induced apoptosis of OS cell lines. After treatment of all three OS cell lines with 15d-PGJ2 at different dose level and durations, cells were co-stained with annexin V and propidium iodide (PI). 15d-PGJ2 significantly induced apoptosis in a dose- and time-dependent fashion (Figure 1C and 1D, respectively). Both these studies indicated that 15d-PGJ2 exerted a cytotoxic effect, inhibiting OS cell growth.

### 15d-PGJ2 induced significant G2/M arrest in OS cell lines

**Figure 2:**
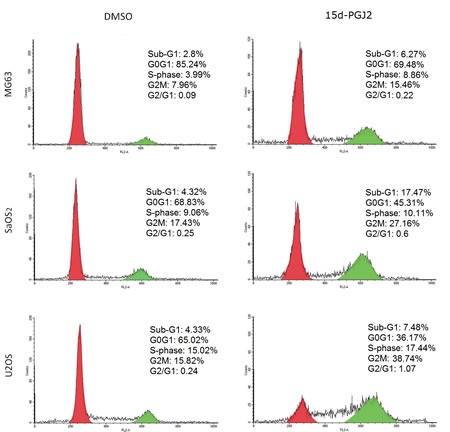
15d-PGJ2 induced significant G2/M arrest in OS cell lines DNA profiles of MG63, SaOS2, and U2OS treated with DMSO or 15d-PGJ2 (20, 10, 10 μmol/L) for 72 h were evaluated by flow cytometry. The percentage of cells in sub-G1, G0G1, G2M, and S phases are shown.

Because PLK1 is a cell cycle regulatory protein, we next examined the effects of 15d-PGJ2 on the cell cycle in OS cells *in vitro*. After treatment with 15d-PGJ2 for 72 h, increased G2/M DNA content was observed in all three OS cell lines (Figure 2), indicating that 15d-PGJ2 could induce a significant G2/M arrest in OS cells.

### 15d-PGJ2 activated ERK but down-regulated both AKT and PKA-PLK1 pathways

**Figure 3:**
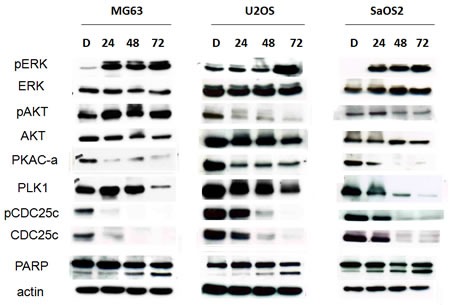
15d-PGJ2 down-regulated both AKT and PKA-PLK1 pathways Western blot analysis of MG63, SaOS2, and U2OS cells treated with DMSO or 15d-PGJ2 (20, 10, 10 μmol/L) for 72 h using antibodies against ERK, p-ERK, AKT, p-AKT, the PKA-PLK1-CDC25 pathway, and PARP.

Since both the PI3K/AKT and PKA-PLK1 signaling pathways have been implicated as targets of 15d-PGJ2, we investigated the effect of 15d-PGJ2 on AKT expression and phosphorylation as well as changes in expression of members of the PKA-PLK1 pathway. As shown in Figure 3, 15d-PGJ2 induced ERK activation, as reported by previous studies [30,39]. On the other hand, 15d-PGJ2 induced a significant time-dependent down-regulation of AKT and p-AKT in U2OS and Saos2 cells, and, to a lesser extent, in MG63 cells. Moreover, 15d-PGJ2 dramatically down-regulated total and phospho- PKA, PLK1, and CDC25 levels in all three cell lines. Cleavage of PARP was also seen after 24–48 h of treatment (Figure 3). Thus, 15d-PGJ2 repressed both the AKT and PKA-PLK1 pathways, thereby inducing apoptosis.

### Both AKT and PKA-PLK1 pathways are critical targets of 15d-PGJ2

**Figure 4:**
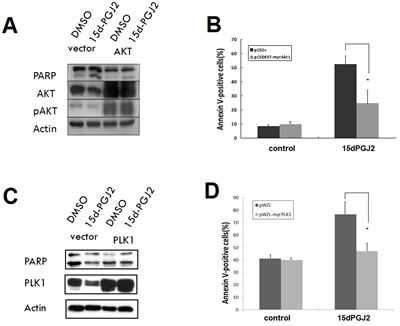
Overexpression of constitutively active AKT or PLK1 can partially reverse the cytotoxic effect of 15d-PGJ2 U2OS cells were transiently transfected with an empty vector or constitutively active AKT or PLK1 expression construct, with or without 15d-PGJ2 (20 μmol/L) for 48 h. (A, C) Immunoblot analysis using corresponding antibodies against (A) AKT or (C) PLK1. (B, D) Apoptosis assay. The percentage of apoptotic cells was determined using Annexin V-FITC/propidium iodide (PI) staining. All data represent the mean ± SD of three independent experiments. *P < 0.05.

To explore the functional significance of AKT and PKA-PLK1 pathway suppression in 15d-PGJ2–mediated cell death, U2OS cells were transiently transfected with a constitutively active AKT or PLK1 expression constructs as well as empty vector controls. Constitutively active AKT or PLK1 expression was confirmed by Western blot analysis (Figure 4A and 4C). Expression of constitutively active AKT or PLK1 partially protected cells from apoptosis induced by 15d-PGJ2 (Figure 4B and 4D). These findings indicate that down-regulation of both AKT and PLK1 plays a functional role in the death of OS cells resulting from treatment with 15d-PGJ2.

### 15d-PGJ2-induced ROS generation in OS cell lines, and cytotoxic effects of 15d-PGJ2 on OS cell lines are ROS-dependent

**Figure 5:**
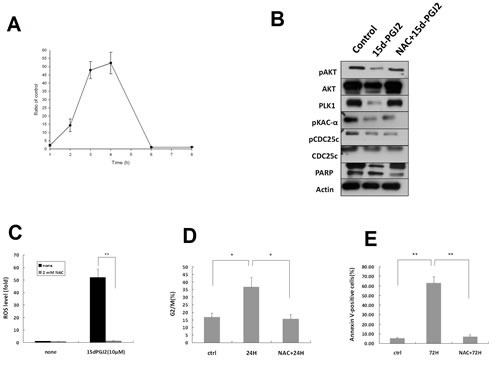
Cytotoxic effects of 15 d-PGJ2 on OS cell lines are ROS-dependent (A) U2OS cells were incubated with 15d-PGJ2 (10 μmol/L) for the indicated time points, labeled with 8OHdG, and analyzed by flow cytometry. ROS level was expressed as an increased ratio in comparison with control. (B) Western blot analysis of USOS cells treated with DMSO or 15d-PGJ2 (20 μmol/L) for 72 h without or with NAC preteatment (2 mM) for 1 h using antibodies against AKT, p-AKT, the PKA-PLK1-CDC25 pathway, and PARP. (C) ROS level of U2OS cells at baseline or treated with 15d-PGJ2 (10 μmol/L) in the absence or presence of NAC (2 mM) for 8 h. (D) G2/M content was evaluated by flow cytometry, and (E) percentage of apoptotic cells was determined using Annexin V-FITC/propidium iodide (PI) staining of U2OS cells at baseline or treated with 15d-PGJ2 (10 μmol/L) with or without NAC (2 mM) for 72 h. All data represent the mean ± SD of three independent experiments. *P < 0.05; **P < 0.01.

ROS generation was considered the major cytotoxic mechanism of 15d-PGJ2 in tumor cell death [32,37]. Therefore, we measured ROS levels in U2OS cell lines exposed to 15d-PGJ2. 15d-PGJ2 induced production of ROS in U2OS cells after 2 h, peaking at 3-4 h (Figure 5A). To investigate a functional relationship between ROS generation and the cytotoxic effect of 15d-PGJ2, U2OS cells were exposed to 15d-PGJ2 in the absence or presence of N-Acetylcysteine (NAC), an antioxidant. As shown in Figure 5B, reduced suppression of the AKT and PKA-PLK1 pathways, as well as PARP degradation was observed in cells treated with 15d-PGJ2 and NAC. In addition, co-treatment of cells with NAC reduced 15d-PGJ2-induced ROS production (Figure 5C) and ameliorated the 15d-PGJ2-induced cell cycle arrest (Figure 5D) and apoptosis (Figure 5E). Thus, 15d-PGJ2 induced ROS generation in OS cell lines, and the cytotoxic effects of 15d-PGJ2 on OS cell lines were mediated by ROS-dependent down-regulation of the PKA-PLK1 and AKT pathways.

### 15d-PGJ2 induced ROS-mediated c-Jun N-terminal kinases (JNK) activation contributes to apoptosis through down-regulation of the AKT and PKA-PLK1 pathways

**Figure 6:**
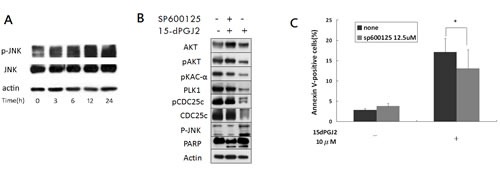
15d-PGJ2-mediated cytotoxicity partially induced through JNK activation (A) The phosphorylation state of JNK was analyzed in U2OS cells treated with 15d-PGJ2 (10 μmol/L) for various time points. (B) U2OS cells were either treated with DMSO, pretreated with SP600125 (12.5 μmol/L for 1 h) and then treated with 15d-PGJ2 (10 μmol/L for 72 h), or treated with 15d-PGJ2 alone. After treatment, the levels of protein expression were determined by Western blot analysis. (C) U2OS cells, with or without pretreatment of SP600125 (12.5 μmol/L for 1 h), were treated with 15d-PGJ2 (10 μmol/L) for 72 h. After treatment, the percentage of Annexin V–positive cells were determined by flow cytometry. All data represent the mean ± SD of three independent experiments. *P < 0.05.

Studies suggest that JNK plays an important role in ROS-induced apoptosis [30,37]. To investigate whether 15d-PGJ2–induced ROS leads to the activation of JNK in OS cells, we examined the phosphorylation state of JNK in OS cells treated with 15d-PGJ2. As shown in Figure 6A, 15d-PGJ2 treatment significantly increased the phosphorylation of JNK. Furthermore, pretreatment with JNK inhibitor, SP600125, for 1 h could prevent the phosphorylation of JNK caused by 15d-PGJ2, and block 15d-PGJ2-induced down-regulation of AKT as well as PKA-PLK1-CDC25 (Figure 6B). SP600125 also inhibited 15d-PGJ2–induced apoptosis (Figure 6C). These results indicate that ROS-mediated JNK activation may in part contribute to apoptosis through down-regulation of AKT and PLK1.

## DISCUSSION

In this study, 15d-PGJ2 inhibited OS cell proliferation by inducing apoptosis. This finding is in agreement with the results of previous studies of 15d-PGJ2 in other cell lines [28,37]. However, the molecular mechanisms underlying the cytotoxic mechanism of 15d-PGJ2–induced apoptosis remained unclear. The present study shows that 15d-PGJ2 induced generation of ROS and activated JNK in OS cells, resulting in the down-regulation of the AKT and PLK1 pathways with subsequent apoptosis.

Several pathways, such as nuclear factor kappa-light-chain-enhancer of activated B cells (NF-κB) [40-42], hypoxia-inducible factor-2α (HIF2α) [43], and AKT [33-37], have been identified as targets for 15d-PGJ2-induced cytotoxicity. AKT is a critical regulator of diverse cellular functions [44], and ROS may activate AKT through phosphatase and tensin homolog (PTEN) inactivation [45]. However, our study disclosed that 15d-PGJ2 down-regulated AKT in OS cells with resultant apoptosis, which is consistent with that of previous studies in other cancer types [34-37].

More importantly, we found that the PKA-PLK1-CDC25 pathway is a target of 15d-PGJ2 on OS cells. A previous study showed that 15d-PGJ2 inhibited PKA in renal proximal epithelial cells [40]. In addition, Zhu et al. [38] showed that exogenous PGD2 and 15d-PGJ2 diminished the viability of human chondrocytes through down-regulation of the PKA-PLK1 pathway [38]. These earlier studies prompted us to explore the possibility of targeting this pathway by 15d-PGJ2 in cancer. In this study, we demonstrated that 15d-PGJ2 down-regulated the PKA-PLK1-CDC25 pathway in OS cell lines. We also showed that constitutive activation of AKT or PLK1 partially reversed the apoptosis induced by 15d-PGJ2. These data confirmed the important role of both AKT and PLK1 in 15d-PGJ2–mediated cytotoxicity of cancer cells.

ROS has been implicated in several oncogenic pathways, such as promoting tumor growth [46], angiogenesis [45], mutagenesis [47,48], and drug resistance [49,50]. On the other hand, ROS-mediated cytotoxicity has also been identified as an important mechanism in some anti-cancer agents [51,52]. Several studies have linked ROS generation with 15d-PGJ2–induced apoptosis [31,32]. In this study, 15d-PGJ2 induced ROS generation in OS cell lines. Moreover, the anti-oxidant, NAC, could reverse the cytotoxic and cell cycle inhibition effects of 15d-PGJ2, confirming the critical role of ROS generation in 15d-PGJ2–induced apoptosis of cancer cell lines.

A previous study suggested that activation of JNK through ROS generation is important for 15d-PGJ2-induced apoptosis [30]. To investigate this hypothesis, we used immunoblot analysis to examine the expression of p-JNK after treatment with 15d-PGJ2. 15d-PGJ2 treatment activated JNK, and SP600125 pretreatment partially inhibited 15d-PGJ2–induced apoptosis. Taken together, these data suggest that activation of JNK through ROS may contribute to down-regulation of AKT and PLK1 to lethality.

In this study, we did not explore other possible mechanisms of 15d-PGJ2-induced cytotoxicity, such as the NF-κB pathway. Therefore, we cannot exclude the possibility that 15d-PGJ2 also inhibits NF-κB in OS. Also, we did not analyze the mechanism of 15d-PGJ2-induced down-regulation of PKA-PLK1-CDC25. Previous studies have shown that 15d-PGJ2 down-regulated AKT at the transcriptional level [35,37]. However, in a recent study analyzing 15d-PGJ2 target proteins, heat shock protein 90 (Hsp90) was found to be one of the targets [53]. Thus, it is also possible that 15d-PGJ2 may inhibit Hsp90 with subsequent degradation of PKA-PLK1-CDC25.

In summary, 15d-PGJ2 inhibited the growth and induced apoptosis of OS cells. 15d-PGJ2 generated ROS, activated JNK, and suppressed the AKT and PLK1 pathways. These results suggested that 15d-PGJ2 may be of therapeutic importance in the treatment of OS, and form the basis for further analysis of its role in animal studies and clinical applications.

### Statement of translational relevance

Polo-like kinase 1 (PLK1) is a key component in cell cycle regulation, and has been identified as a potential target of osteosarcoma (OS). On the other hand, 15-deoxy-Δ12, 14-prostaglandin J2 (15d-PGJ2) has been found to mediate shear-induced chondrocyte apoptosis via protein kinase A (PKA)-dependent regulation of PLK1. In the current study, we explored the cytotoxic effect and mechanism of 15d-PGJ2 in an *in vitro* model of OS. In OS cell lines, 15d-PGJ2 induced a significant cytotoxic effect, including G2/M arrest and apoptosis. Moreover, the cytotoxicity of 15d-PGJ2 resulted from ROS-mediated, JNK-dependent down-regulation of both the AKT and PKA-PLK1 pathways in OS. The current study revealed a unique mechanism of 15d-PGJ2 against OS, and its efficacy in xenograft models and clinical usages deserved further exploration.

## MATERIALS AND METHODS

### Cell lines and reagents

Three OS cell lines, U2OS, MG63 and SaOS2, were chosen as our *in vitro* study model. They were kept in the DMEM or IMDM base media with 10% fetal bovine serum (FBS). 15d-PGJ2 was purchased from Calbiochem (San Diego, CA, USA). The following antibodies were used for immunoblotting: PKA C-α (Cell Signaling, Danvers, MA; #4782; 1:1000); PLK1 (Cell Signaling #4513; 1:1000); AKT (Cell Signaling #9272; 1:2000); p-AKT (Cell Signaling #9271; 1:1000); Cdc25c(5H9) (Cell Signaling #4688; 1:1000); P-cdc25c (Ser216) (Cell Signaling #4901; 1:1000); PARP (Cell Signaling #9542; 1:1000), and actin (Abs 24-100; 1:50000). The anti-8-hydroxy-2'-deoxyguanosine (8OHdG) antibody (Santa Cruz, #sc-66036; 1:200) and anti-mouse conjugated fluorescein isothiocyanate (FITC) antibody (Jackson ImmunoResearch West Grove, PA; 1:400) were used for 8OHdG detection.

### Analysis of cell viability

Cells were seeded in triplicate 96-well plates in 100 μL complete media at a density of 2000-20000 cells per well. On the next day, drugs were added at different concentrations with variable times. Then, 10 μL 3-(4,5-dimethylthiazol-2-yl)-2,5-diphenyltetrazolium bromide (MTT; Sigma-Aldrich) solution was added to the wells and the plates were incubated for an additional 4 h at 37°C. A detergent solution (200 μL/well) was next added and mixed thoroughly to dissolve the dark-blue crystals. Absorbance of the converted dye was measured spectrophotometrically using a microplate reader (Vmax, Molecular Devices, Sunnyvale, CA) at 570 nm (test) and 650 nm (reference). Cell survival was calculated as percentage of MTT inhibition as follows: % survival = (mean experimental absorbance/ mean control absorbance) × 100 [54].

### Apoptosis assessment by annexin V staining

Drug-induced apoptosis was measured using annexin V-fluorescein isothiocyanate (Annexin V-FITC) and PI co-staining using an Annexin V-FITC apoptosis detection kit (BD Pharmingen, San Diego, CA). After 15d-PGJ2 or DMSO treatment, cells were washed and resuspended in 100 μL staining solution (containing Annexin V-FITC and PI in a HEPES buffer). After incubation in dark and at room temperature for 15 min, cells were diluted in 400 μL of 1x binding buffer, and the percentages of apoptotic cells were analyzed by flow cytometry using a FACS Calibur (Becton Dickinson & Co., Oxford, CA, USA) and CellQuest software (Becton Dickinson & Co.).

### Cell cycle analysis

After 15d-PGJ2 or DMSO treatment, OS cells were trypsinized and fixed in 99% ethanol at−20°C for 2 h, washed and re-suspended in 420 μL PBS. Subsequently, samples were first incubated with RNase A (Sigma) (50 μL of a 10 mg/mL solution) at 37°C for 30 min, and then PI (20 μL of a 0.2 mg/mL solution) at room temperature for 10 min. DNA content was analyzed by flow cytometry using a FACS Calibur (Becton Dickinson & Co.) and CellQuest software (Becton Dickinson & Co.) [55].

### Western blot analysis

Cell extracts were prepared with RIPA Lysis and Extraction Buffer (Thermo Scientific, Rockford, IL) containing a protease and phosphatase inhibitor cocktail (1:100 dilution; Thermo Scientific). Protein concentrations were determined using the BCA Protein Assay Kit (Thermo Scientific). Aliquots of protein lysates were electrophoretically separated on sodium dodecyl sulfate–polyacrylamide gels and transferred to polyvinylidene fluoride membranes (Millipore, Billerica, MA), which were blocked with 5% blotting grade milk (Bio-Rad, Hercules, CA) in TBST (20mM Tris-HCl [pH 7.6], 137mM NaCl, 1% Tween 20). Membranes were then probed with the indicated primary antibodies, reacted with corresponding secondary antibodies, and were detected using an enhanced chemiluminescence system (Millipore) and X-ray films.

### Transfection of the Myr-Akt or PLK1 vector

In order to explore the critical function of AKT or PLK1 in 15d-PGJ2-induced cytotoxicity, U2OS cells (7×10^5^) were transfected with either 8 μg of myc-tagged myristoylated Akt expression vector (pCDEST-myrAkt) or pWZL NeoMyrFlag PLK1 expression vector or their corresponding empty vector (pCS2+ or pWZL-Neo-Myr-Flag-DEST) (all purchased from Addgene, Cambridge, MA, USA) using LipofectAMINE (Invitrogen, Carlsbad, CA, USA) according to the manufacturer's procedure. After transfection, cells were cultured in 10% FBS–supplemented IMDM for 24 h and then subjected to 0.1% DMSO or 15d-PGJ2 treatment.

### Measurement of ROS

The formation of the 8-hydroxy-2'-deoxyguanosine (8OHdG) base lesion, which is a biomarker for oxidative stress [56], was measured as previously described [57], with modification. Briefly, cells were trypsinized and harvested, washed twice with PBS and fixed by 1% paraformaldehyde at room temperature. The fixed cells were then washed with PBS, permeabilized by adding 0.1% Triton X100 for 15 min at room temperature, and washed with PBS again. Next, a blocking solution consisting of 1% BSA in PBS was added for 1 h at 37°C with gentle shaking, and cells were incubated with anti-8OHdG antibody for 1 h at 37°C after washing. Cells were washed again with PBS, and anti-mouse conjugated FITC antibody was then added and incubated for 1 h. Flow cytometry was used to detect the immunofluorescence.
